# Knee Osteoarthritis (KOA) Severity Influences Proximal Femoral Biomechanics and Predicts Pertrochanteric Fracture Instability: A Retrospective Radiographic Study

**DOI:** 10.3390/medicina62030469

**Published:** 2026-03-01

**Authors:** Seyed Ali Hashemi, Bahram Abedini, Hossein Hosseini, Shayan Yousufzai, Christos Koutserimpas, Georgi P. Georgiev, George Tiantafyllou, Eva Diomidous, George Tsakotos, Ioannis Paschopoulos, Fotios Kantas, Maria Piagkou

**Affiliations:** 1 Department of Orthopedic Surgery, Chamran Hospital, Shiraz University of Medical Sciences, Shiraz 71348, Iran; erfanmydream@yahoo.com (S.A.H.); b9141864317@gmail.com (B.A.); 2 Student Research Committee, Shiraz University of Medical Sciences, Shiraz 71348, Iran; hossein.husseini@yahoo.com (H.H.); yousefzaishayan@gmail.com (S.Y.); 3 School of Rehabilitation Health Sciences, University of Patras, 26504 Patras, Greece; chrisku91@hotmail.com; 4 Department of Orthopedics and Traumatology, University Hospital Queen Giovanna-ISUL, Medical University of Sofia, 1527 Sofia, Bulgaria; georgievgp@yahoo.com; 5 Department of Anatomy, School of Medicine, Faculty of Health Sciences, National and Kapodistrian University of Athens, 11527 Athens, Greece; georgerose406@gmail.com (G.T.); evadiomidous@gmail.com (E.D.); gtsakotos@gmail.com (G.T.); johnpascho@gmail.com (I.P.); f.d.kantas@gmail.com (F.K.)

**Keywords:** knee osteoarthritis, Kellgren–Lawrence, intertrochanteric fracture, fracture stability, lateral wall thickness, AO/OTA classification, hip fracture biomechanics

## Abstract

*Background and Objectives:* Intertrochanteric hip fractures (ITFs) are common in older adults and frequently coexist with knee osteoarthritis (KOA). Although both conditions share key biomechanical risk factors, the specific relationship between KOA severity and ITF stability has not been well defined. Recent evidence suggests that degenerative knee changes may alter lower-limb load distribution and increase susceptibility to unstable fracture patterns. This study evaluated whether KOA severity, graded using the Kellgren–Lawrence (KL) system, is associated with ITF stability according to the 2018 AO/OTA classification. *Materials and Methods:* A retrospective observational study was conducted on 138 patients with IHFs treated between 2018 and 2023. KOA severity was assessed using KL grades I–IV on non-weight-bearing anteroposterior knee radiographs. Lateral wall thickness (LWT) was measured using the Hsu method, with <20.5 mm indicating fracture instability. Statistical analyses included correlation, linear regression, logistic regression, and receiver operating characteristic (ROC) curve analysis to examine the association between KL grade and fracture stability. *Results:* Among 138 patients, 98 (71.0%) had unstable ITFs. Advanced KOA was significantly more common in the unstable group (KL III 45.9%, KL IV 48.0%; *p* < 0.001). KL grade showed a significant inverse correlation with LWT (Pearson’s r = −0.394, *p* < 0.001). Each one-grade increase in KL severity was associated with a 3.8 mm reduction in LWT (*p* < 0.001). In multivariable logistic regression, KL grade remained an independent predictor of fracture instability (adjusted OR = 4.9, 95% CI: 2.8–8.8, *p* < 0.001), whereas age and comorbidities were not significant. ROC analysis demonstrated good discriminatory power (AUC = 0.79). A KL ≥ III threshold achieved 95% sensitivity and 56% specificity for predicting instability. *Conclusions:* Higher KOA severity is strongly associated with unstable ITF patterns. KL grade independently predicts instability and may serve as a simple, accessible radiographic indicator of biomechanical vulnerability and fracture risk in older adults. Incorporating KOA severity into the preoperative evaluation may enhance risk stratification, guide selection of fixation strategy, and support individualized rehabilitation planning.

## 1. Introduction

Hip fractures (HFs) represent one of the most serious musculoskeletal injuries in older adults and are associated with substantial morbidity, mortality, and functional decline [1]. Intertrochanteric hip fractures (ITFs), in particular, are common in frail and osteoporotic populations following low-energy falls. Pertrochanteric fractures refer to fractures occurring in the trochanteric region of the proximal femur, involving the area around the greater and lesser trochanters [2]. In clinical usage, pertrochanteric fractures largely overlap with ITFs, which are formally defined as fractures extending between the greater and lesser trochanters. While the terms are often used interchangeably, ITF is the preferred term in classification systems such as AO/OTA [3].

HFs account for more than 1.6 million cases worldwide each year, and their incidence is projected to increase substantially as the population ages. Recent studies indicate that individuals with prior hip interventions, such as arthroplasty, remain at heightened risk of contralateral or periprosthetic fractures, underscoring the continuing global health burden of these injuries [4].

Knee osteoarthritis (KOA) is also highly prevalent in aging individuals and is a major contributor to disability, impaired gait, and altered lower-limb mechanics [5,6]. Because both conditions share key risk factors—including advanced age, compromised bone quality, and gait abnormalities- their coexistence is frequent [7,8].

Despite this overlap, the potential influence of KOA severity on IHF morphology and stability remains poorly understood. KOA leads to progressive structural degeneration, including malalignment, joint space narrowing, proprioceptive deficits, reduced quadriceps strength, and diminished shock absorption [3,9]. Given the interdependence of the lower-limb kinetic chain, degenerative alterations at the knee may modify force vectors transmitted to the proximal femur during gait or a fall [10]. These changes may predispose individuals to distinct, potentially more unstable fracture configurations [11,12,13], particularly given that lateral-wall integrity—critical to fracture stability—can be compromised by altered biomechanical loading [14].

Growing clinical evidence supports this biomechanical relationship. Polat et al. (2025) [15] reported that higher Kellgren–Lawrence (KL) grades were associated with unstable AO/OTA type 2–3 fractures. Similar findings were described by Davut and Kalacı (2022) [16], who observed a higher prevalence of KOA among patients with trochanteric fractures. KOA may also influence postoperative outcomes; Lv et al. (2023) [17] found that KOA in elderly IHF patients was associated with slower healing and poorer functional recovery. While prior studies of hip osteoarthritis (HOA) have shown that degenerative hip morphology influences fracture type [18,19], the impact of KOA on ITF stability has received limited attention. Other pathological processes—such as brucellosis-induced avascular necrosis—demonstrate how joint abnormalities can alter load bearing and fracture behavior [20].

Given these biomechanical considerations and the limited evidence base, a more detailed exploration of the relationship between KOA severity and proximal femoral fracture stability is warranted. Accordingly, this retrospective study aimed to determine whether KOA severity, graded using the KL system, is associated with ITF stability according to the 2018 AO/OTA classification, and to evaluate whether degenerative changes at the knee can serve as an independent biomechanical factor contributing to fracture instability.

## 2. Materials and Methods

This retrospective observational study included patients diagnosed with ITFs who were treated at affiliated hospitals of the Shiraz University of Medical Sciences between January 2018 and December 2023. The study protocol was approved by the Institutional Ethics Committee of the Shiraz University of Medical Sciences (Approval No. IR.SUMS.MED.REC.1402.054), and all procedures adhered to the Declaration of Helsinki and its subsequent amendments. All patients aged ≥ 18 years who presented with hip fractures during the study period were screened for eligibility. Of the 217 individuals initially assessed, 138 (63.6%) met the inclusion criteria and were included in the final analysis.

Inclusion criteria: Eligible patients were required to be 18 years of age or older, have a confirmed diagnosis of ITF based on clinical evaluation and standardized radiographic assessment, and possess knee radiographs of sufficient quality to allow KL grading.

Exclusion criteria: Patients were excluded if radiographic data were absent or of inadequate quality for accurate KL grading or lateral wall thickness (LWT) measurement, if they had a history of knee arthroplasty, major reconstructive knee surgery, or previous femoral fractures altering native anatomy, or if demographic or clinical data were incomplete.

Radiograph availability and methodological considerations. All imaging was performed during the acute post-fracture phase. Because patients were unable to tolerate standing due to pain and immobility, load-bearing knee radiographs could not be obtained. Consequently, only non-load-bearing anteroposterior knee radiographs were available, and no pre-injury knee radiographs existed for any patient. This represents a significant methodological limitation. However, orthopedic surgeons confirmed that KL grades III and IV can be reliably identified on non-weight-bearing images because of characteristic features such as large osteophytes, pronounced subchondral sclerosis, and distinct osseous deformity—features that remain visible even without weight-bearing stress. After applying all criteria, 138 patients with complete clinical and radiographic datasets were included in the study cohort.

It should be noted that radiographic data were collected exclusively during the acute admission period, without any pre-fracture imaging. Therefore, all KOA assessments reflect structural degenerative status at the time of injury rather than pre-existing baseline conditions.

Data were extracted from electronic medical records using a structured checklist and were verified through the Shiraz University of Medical Sciences “Infinite” database. Collected variables included demographic characteristics; anthropometric data (age, sex, height, weight, and body mass index [BMI]); comorbidities such as hypertension, diabetes mellitus, heart disease, and hyperlipidemia; and radiographic parameters describing hip and knee morphology.

Osteoporosis-related parameters, including bone mineral density (BMD), and detailed fall-mechanism characteristics (e.g., fall direction, velocity, or impact point), were not incorporated as covariates due to inconsistent or missing documentation in the retrospective dataset. This limitation is common in non-prospective orthopedic research.

**Figure 1:**
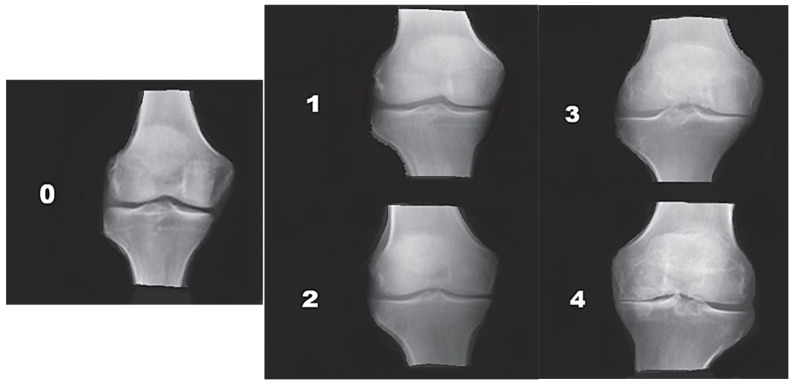
Kellgren–Lawrence (KL) radiographic grades of knee osteoarthritis. Representative anteroposterior knee radiographs demonstrating the five KL grades of osteoarthritis, from Grade 0 (no OA) to Grade 4 (severe OA). Grade 0: No radiographic features of osteoarthritis (normal joint appearance). Grade 1: Doubtful joint space narrowing with possible osteophytic lipping. Grade 2: Definite osteophyte formation with possible joint space narrowing. Grade 3: Multiple osteophytes, definite joint space narrowing, subchondral sclerosis, and potential bone contour deformity. Grade 4: Large osteophytes, severe joint space narrowing, pronounced subchondral sclerosis, and definite deformity of bone ends.

KL Grading. The severity of KOA was assessed using the Kellgren–Lawrence (KL) classification, which categorizes radiographic osteoarthritic changes into four grades (Figure 1) [15]. KL grade was treated as an ordinal variable (I–IV) for correlation and regression analyses to reflect its progressive nature. Grade I: doubtful joint-space narrowing and possible osteophytic lipping. Grade II: definite osteophytes with possible joint-space narrowing. Grade III: multiple osteophytes, marked joint-space narrowing, and subchondral sclerosis. Grade IV: large osteophytes, severe joint-space narrowing, pronounced subchondral sclerosis, and deformity of bone contours.

**Figure 2:**
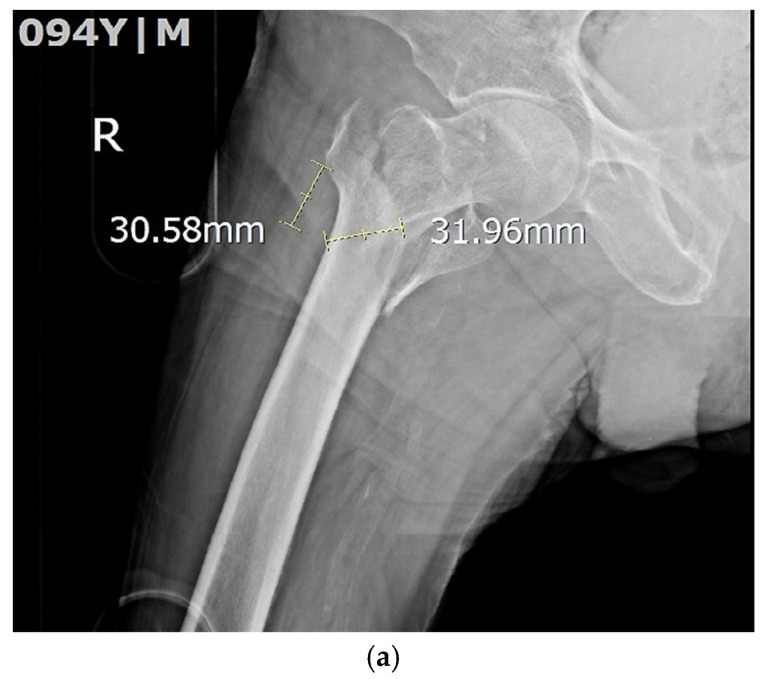

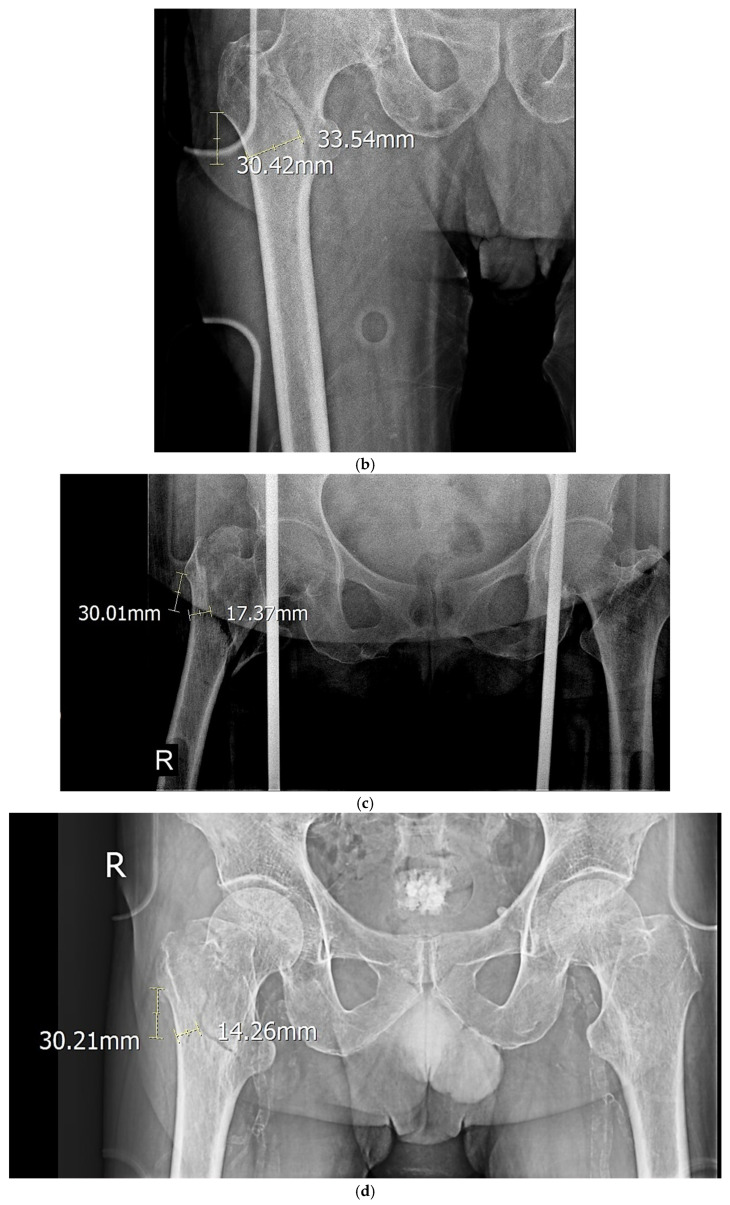

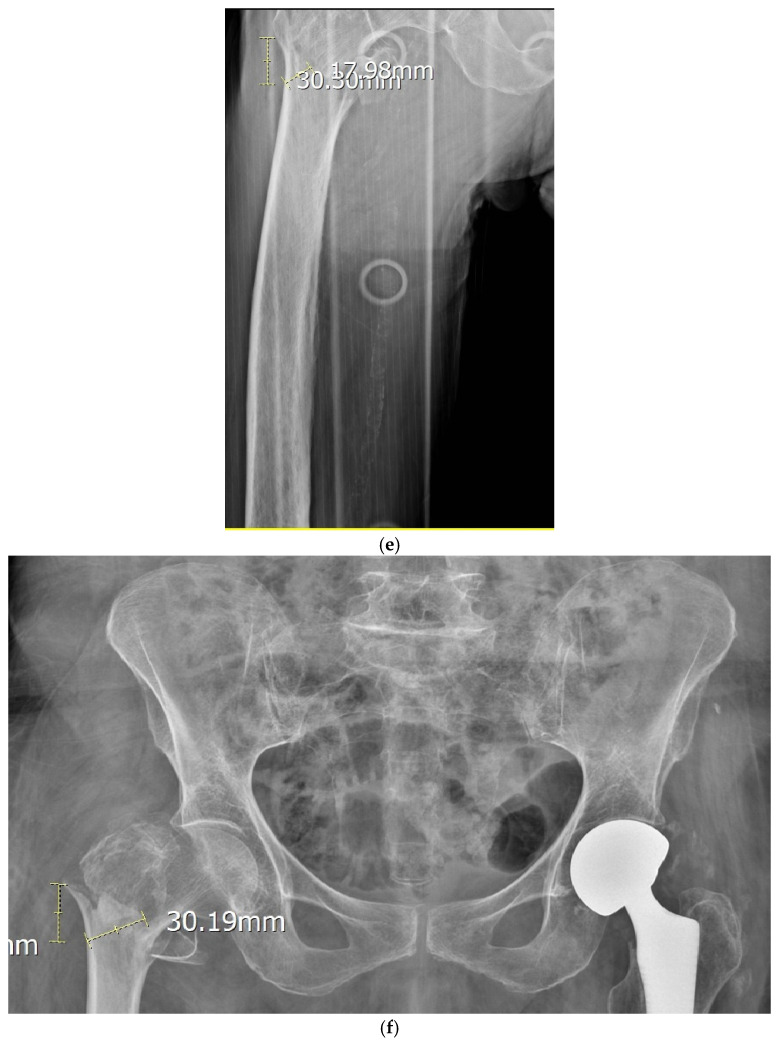
(**a**) Unstable pertrochanteric fracture: measurement of lateral femoral wall thickness (LWT). Anteroposterior radiograph of the right hip demonstrating the standard method for LWT assessment. A point 3 cm distal to the innominate tubercle of the greater trochanter is identified, and a 135° reference line is constructed to approximate the cephalomedullary nail trajectory. The cortical thickness along this line measures 31.96 mm, indicating a stable lateral wall (≥20.5 mm). The proximal measurement of 30.58 mm corresponds to the cortical dimensions. (**b**) Stable peritrochanteric fracture with preserved lateral wall thickness. Radiograph demonstrates LWT of 33.54 mm, well above the threshold for stability. Cortical continuity and thickness are clearly visualized, consistent with AO/OTA stable fracture morphology. The adjacent measurement of 30.42 mm provides anatomical reference. (**c**) Unstable pertrochanteric fracture demonstrating compromised lateral wall thickness (LWT). The LWT measured along the 135° reference line is 17.37 mm, which falls below the accepted cutoff of 20.5 mm for lateral wall insufficiency. This measurement indicates high instability risk, in agreement with AO/OTA type 31A2–A3 patterns. The proximal measurement of 30.01 mm is shown for contextual comparison. (**d**) Pertrochanteric fracture with markedly reduced lateral wall thickness (LWT). Radiograph showing LWT of 14.26 mm, reflecting advanced cortical compromise. This degree of thinning is characteristic of unstable fracture patterns associated with collapse risk and medialization during load bearing. (**e**) Measurement of a deficient lateral femoral wall in an unstable fracture. The measured lateral wall thickness (LWT) is 17.98 mm, indicating insufficient cortical support. Such values are known predictors of postoperative lateral wall fracture and mechanical failure risk, consistent with published thresholds. (**f**) Unstable intertrochanteric fracture (ITF) in a patient with contralateral hip arthroplasty. Radiographic lateral wall thickness (LWT) measurement is illustrated on the fractured side, while the contralateral hip arthroplasty component is visible. This image demonstrates cortical contour assessment in a complex anatomical setting. If LWT < 20.5 mm on final measurement, this represents an unstable pattern; otherwise, it may serve as a comparative anatomical reference.

Lateral Wall Thickness (LWT) Measurement. Fracture stability was assessed according to the 2018 AO/OTA classification, with specific emphasis on LWT. LWT was measured following the method described by Hsu et al. [14]. The innominate tubercle of the greater trochanter was identified, a point 3 cm distal along the lateral cortex was marked, and a 135° reference line was drawn relative to the femoral shaft axis. Cortical thickness along this line represented the LWT. Representative examples are shown in Figure 2.

An LWT ≥ 20.5 mm was defined as stable, whereas an LWT < 20.5 mm was defined as unstable. This cutoff was based on biomechanical validation by Hsu et al. [14] (2013), who demonstrated that LWT < 20.5 mm significantly increased the risk of postoperative lateral wall fractures and mechanical failure. The same threshold has since been consistently supported by multiple trauma studies [15] and remains the accepted clinical benchmark for defining lateral wall insufficiency. All radiographs were obtained at admission. No pre-fracture knee radiographs were available. Two fellowship-trained orthopedic surgeons, blinded to patient clinical information, independently assessed all KL grades and LWT measurements. Inter-rater reliability was excellent for both KL grading (weighted κ = 0.84) and LWT measurement (intraclass correlation coefficient [ICC] = 0.92). The primary objective of the study was to determine whether KOA severity (KL grade) was independently associated with ITF stability.

## 3. Results

**Figure 3:**
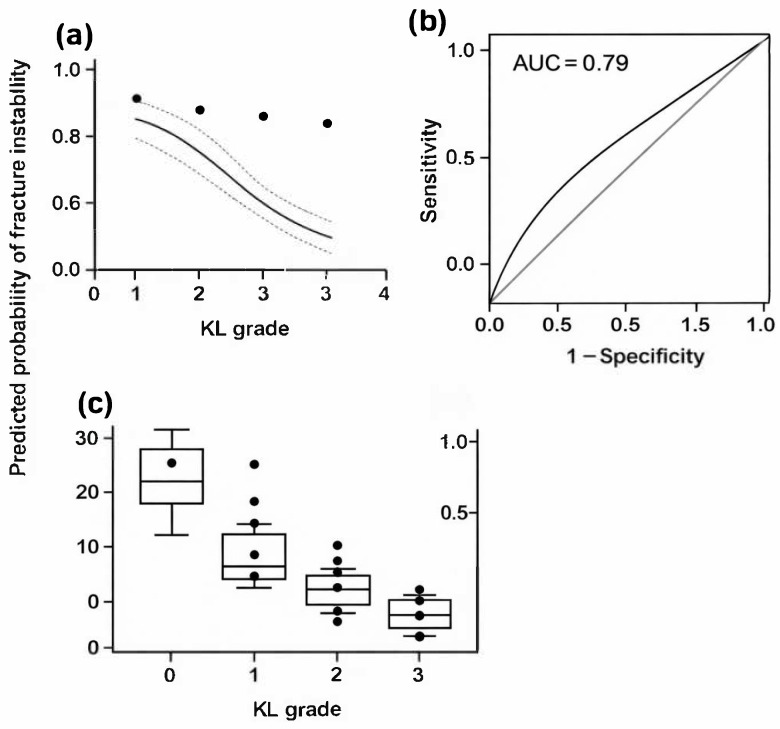
Association between Kellgren–Lawrence grade and fracture instability. (**a**) Binary logistic regression model illustrating the predicted probability of intertrochanteric fracture instability according to Kellgren–Lawrence grade (KLG I–IV) of knee osteoarthritis (KOA). The solid line represents the predicted probability of instability, and the dotted lines indicate the 95% confidence interval (CI) of the model fit. Black dots represent the observed proportion of unstable fractures within each KL grade category. (**b**) Receiver operating characteristic (ROC) curve demonstrating the discriminatory performance of KL grade for predicting fracture instability (AUC = 0.79). The diagonal gray line represents the reference line for random classification. (**c**) Box-and-whisker plot showing the distribution of lateral wall thickness (LWT) across KL grades. The box represents the interquartile range (IQR), the horizontal line indicates the median, whiskers denote the minimum and maximum values, and individual dots represent outliers.

**Figure 4:**
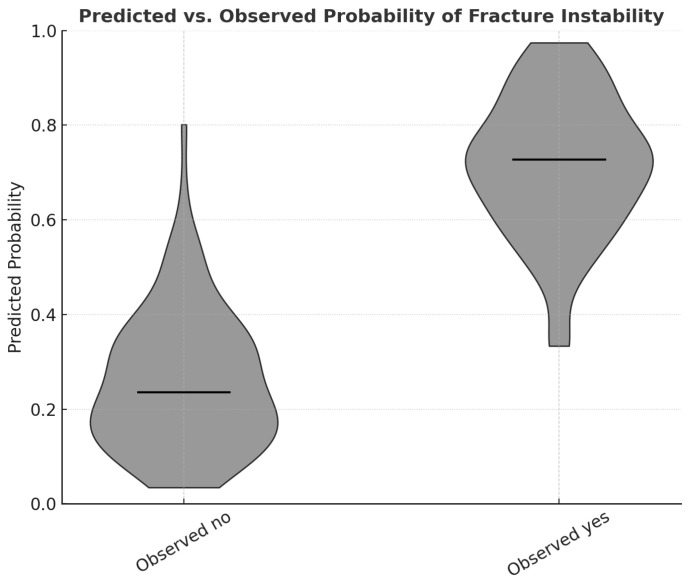
Predicted vs. observed probability of intertrochanteric fracture instability. Violin plots illustrating the distribution of predicted probabilities from the logistic regression model according to observed fracture stability status. The left violin represents observed stable fractures, characterized by lower predicted instability probabilities. The right violin represents observed unstable fractures, characterized by higher predicted probabilities. The horizontal dashed line within each violin indicates the median predicted probability for the respective group.

**Figure 5:**
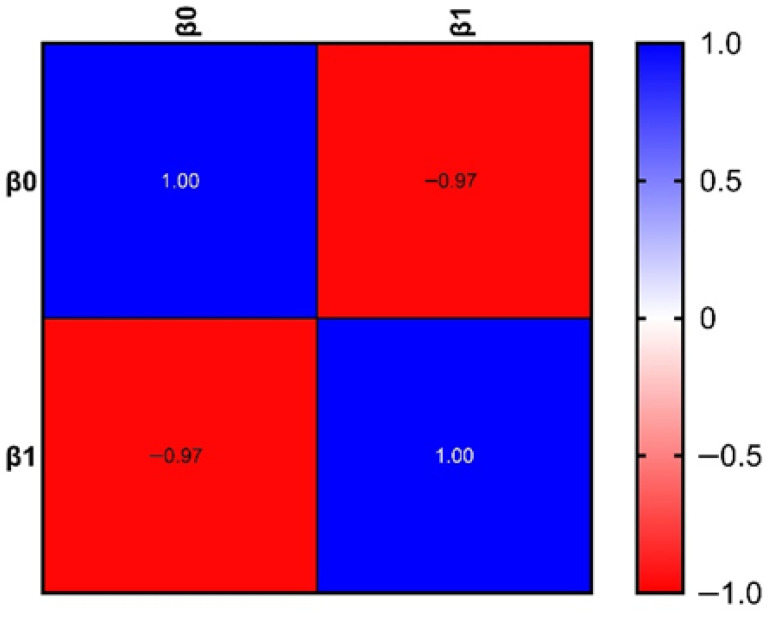
Correlation matrix of logistic regression coefficients. Heatmap illustrating the correlation matrix between regression parameters (β_0_: intercept; β_1_: KL grade coefficient) in the logistic regression model. Color intensity represents the magnitude and direction of correlation (blue = positive; red = negative). A strong negative correlation (r = −0.97) is observed between β_0_ and β_1_, reflecting the expected parameter interdependence within the fitted model.

A total of 138 patients with ITFs were included in the final analysis. Among them, 98 patients (71.0%) presented with unstable fracture patterns, while 40 patients (29.0%) exhibited stable patterns. The mean age of the study population was 72.3 ± 16.5 years, and 57.2% were male. Patients with unstable fractures were significantly older than those with stable fractures (75.2 ± 13.0 vs. 65.6 ± 21.4 years, *p* = 0.011). A higher proportion of males was observed in the stable group (72.5%) than in the unstable group (51.0%; *p* = 0.021). Other baseline variables, including weight, height, BMI, and comorbidities such as hypertension, diabetes mellitus, and heart disease, showed no significant differences between the two groups (Table 1). These demographic findings confirm that while age differed between the two groups, other potential confounders—including BMI and systemic comorbidities—were evenly distributed, supporting the comparability of the study populations prior to statistical adjustment. The severity of KOA, as assessed by the KL grading system, showed a strong and graded association with ITF stability. In the stable fracture group, most patients demonstrated mild-to-moderate KOA (KL grade II, 57.5%). In contrast, the unstable fracture group showed a predominance of advanced degenerative changes, with KL grade III in 45.9% and KL grade IV in 48.0% of cases (*p* < 0.001). A significant linear trend was observed, indicating a clear stepwise increase in instability frequency with higher KL grades (linear-by-linear association: χ^2^ = 41.05, *p* < 0.001). The mean LWT was significantly lower among patients with unstable fractures compared with those with stable fractures (13.2 ± 5.2 mm vs. 29.9 ± 5.9 mm, *p* < 0.001). Because the LWT distribution was non-normal (Kolmogorov–Smirnov *p* < 0.001), the result was further verified using a Mann–Whitney U test (*p* < 0.001), confirming robustness. As illustrated in Figure 3, a clear and progressive decrease in LWT was evident with increasing KOA severity, particularly among unstable fractures, demonstrating a dose–response effect between degenerative grade and cortical thinning. Correlation analyses revealed a moderately strong inverse relationship between KL grade and LWT across the study cohort (Pearson’s r = −0.394, *p* < 0.001). Nonparametric correlations yielded consistent findings (Spearman’s ρ = −0.403, *p* < 0.001; Kendall’s τ = −0.316, *p* < 0.001), demonstrating the robustness of the association (Table 2). Linear regression analysis further indicated that each one-grade increase in KL severity corresponded to an average 3.8 mm decrease in LWT (β = −3.8, 95% CI: −5.0 to −2.6, *p* < 0.001), confirming a graded biomechanical relationship between KOA severity and cortical integrity. In the multivariate logistic regression model, KL grade emerged as an independent predictor of fracture instability (adjusted OR = 4.9, 95% CI: 2.8–8.8, *p* < 0.001). Notably, after adjustment for age, sex, BMI, and comorbidities, no other covariate retained statistical significance in the final model, suggesting that KOA severity independently contributes to the likelihood of an unstable fracture pattern. The model exhibited good calibration (Hosmer–Lemeshow *p* = 0.25) and moderate explanatory power (Nagelkerke R^2^ = 0.21) (Table 3). The predictive performance of KL grade as a single diagnostic variable was strong (Model 2), achieving an area under the ROC curve (AUC) of 0.79 (95% CI: 0.70–0.88). A KL grade ≥ III was identified as the optimal threshold for predicting fracture instability, yielding 95% sensitivity and 56% specificity (Youden Index = 0.51). Overall classification accuracy was 84.1%, with a positive predictive value (PPV) of 80.0% and a negative predictive value (NPV) of 85.2% (Table 4). Figure 3 presents the ROC curve, highlighting the discriminative capability of the KL grade in identifying unstable fractures. Collectively, these findings suggest that greater KOA severity is consistently associated with thinner lateral femoral walls and a significantly higher probability of unstable fracture configurations. Although causality cannot be established due to the study’s retrospective design, the results robustly support the hypothesis that advanced degenerative changes in the knee biomechanically influence proximal femoral stability, highlighting the kinetic interdependence of the knee and hip joints. As shown in Figure 4, the distribution of predicted probabilities differs substantially between the observed stable and unstable groups, supporting the discriminatory performance of the logistic regression model. Multicollinearity diagnostics were performed prior to regression modeling. As shown in Figure 5, the correlation matrix of regression coefficients shows a strong inverse relationship between β_0_ and β_1_ (r = −0.97), reflecting the expected mathematical relationship between the intercept and slope parameters in the logistic regression model.

## 4. Discussion

The present study demonstrated a strong, consistent correlation between the severity of KOA, as graded by the KL system, and ITF stability. Patients with advanced KOA were markedly more likely to exhibit unstable fracture patterns compared to those with mild or moderate degenerative changes. KL grade remained an independent predictor of instability in multivariable analyses, suggesting that degenerative knee changes exert a measurable biomechanical influence on proximal femoral stability during falls. Although causality cannot be established from this retrospective design, the consistency and strength of the observed relationship emphasize the importance of KOA severity as a biomechanical factor influencing fracture morphology [5,6].

Importantly, while patients with unstable fractures were significantly older, age did not retain independent predictive value after adjustment in the regression model. This finding directly addresses concerns that the observed association could simply reflect age-related degeneration. Instead, it suggests that the structural and functional consequences of KOA—rather than chronological age—are more relevant to the biomechanical predisposition for fracture instability.

Comparison with Previous Literature: The results of this investigation are consistent with earlier studies linking KOA severity to unstable proximal femoral fractures. Polat et al. (2025) [15] observed that patients with advanced KOA were more likely to exhibit AO/OTA type II–III fractures than those with milder grades. Similarly, Davut and Kalacı (2022) [16] reported that KOA was significantly more prevalent among individuals with intertrochanteric fractures than among those with femoral neck or subtrochanteric fractures, suggesting a biomechanical influence of degenerative knee changes on fracture type. These results align with biomechanical and gait analyses demonstrating that KOA profoundly alters load distribution, stride dynamics, and limb alignment [9]. Hyodo et al. (2017) [10] emphasized the functional interdependence of the hip, knee, and ankle joints within the kinetic chain, indicating that degenerative changes in one segment affect the entire limb’s mechanics [21]. Broader epidemiological studies also link KOA with increased fall risk [7], and studies of fall biomechanics confirm that impact direction, momentum, and velocity substantially influence hip fracture severity and pattern [11,12,13]. Furthermore, prior investigations into hip morphology and osteoarthritis (OA) [14,18,22] and recent genetic analyses [23] have shown that structural joint variations and degenerative changes contribute to fracture susceptibility.

The present study expands upon this literature by demonstrating that KOA severity not only affects fracture type but also predicts the degree of fracture instability—a distinct and clinically important parameter.

Biomechanical Interpretation: The mechanisms linking KOA to fracture instability are likely multifactorial. KOA produces progressive joint malalignment, osteophyte formation, cartilage loss, and subchondral sclerosis [6,24]. These degenerative changes alter the transmission of mechanical loads through the lower limb, thereby modifying the force vectors acting on the proximal femur during gait or falls [10]. Neuromuscular deficits commonly associated with KOA—such as quadriceps weakness, impaired proprioception, balance instability, and fear of falling—further affect fall mechanics [25,26,27]. These biomechanical and neuromuscular alterations may change the angle and magnitude of ground impact, increasing the likelihood of unstable fracture patterns during a fall. Analogous mechanisms have been reported in other degenerative and inflammatory joint conditions: for example, brucellosis-induced avascular necrosis has been shown to weaken bone integrity and predispose complex fracture morphologies [20]. Together, these findings support the concept that chronic joint pathology alters load-bearing behavior, rendering the proximal femur more vulnerable to unstable fracture configurations when subjected to impact.

Clinical Implications: The demonstrated relationship between KOA severity and IHF instability carries significant surgical and rehabilitative implications. Preoperative assessment of knee radiographs can provide valuable predictive information, enabling surgeons to anticipate unstable fracture morphology and plan accordingly. In patients with advanced KOA (KL grade ≥ III), more robust fixation strategies or even consideration of arthroplasty may be justified when lateral wall insufficiency is suspected. Specifically, surgeons might select longer or static-locking cephalomedullary nails, employ augmentation techniques, and prioritize preservation of the lateral wall. Moreover, awareness of the biomechanical vulnerability associated with advanced KOA may inform tailored postoperative protocols, such as delayed or graded weight-bearing and more intensive physiotherapy programs. Lv et al. (2023) [17] similarly observed inferior postoperative outcomes and slower recovery in IHF patients with concomitant KOA, reinforcing the need for individualized rehabilitation. Incorporating KOA grading into preoperative risk assessment may therefore enhance surgical planning, improve patient counseling, and optimize postoperative management—complementing traditional parameters such as bone mineral density (BMD).

**Methodological Considerations**: Several methodological factors warrant discussion. Because patients were assessed acutely after fracture, weight-bearing radiographs could not be obtained. Although KL grades III and IV can be confidently evaluated on non-weight-bearing images [22], earlier grades may be underestimated. Similarly, LWT measurements were derived from static radiographs, which may not perfectly represent dynamic loading conditions. Nevertheless, inter-rater reliability remained excellent, consistent with previous validation studies [14,28].

The absence of bone mineral density (BMD) and detailed fall-mechanism data is a notable limitation, reflecting the dataset’s retrospective nature and challenges in extracting these variables from routine records. Other unmeasured confounders—such as lower-limb strength, frailty, or gait parameters—may also contribute to fracture risk. The single-center design, while methodologically consistent, limits external generalizability. To ensure model robustness, multicollinearity diagnostics were conducted prior to regression; all predictors showed low intercorrelation (VIF < 2), confirming the model’s stability.

Limitations of the Study: This study’s limitations should be interpreted in context. Because imaging was performed during the acute phase, pre-injury knee radiographs were unavailable. While orthopedic surgeons could reliably classify KL grades III and IV based on osteophyte formation and sclerosis, early-stage KOA grading may have been less precise. LWT measurements, although reliable, were derived from non-weight-bearing images and may not fully capture functional cortical loading. Additionally, the lack of BMD, fall mechanics, and neuromuscular data precluded comprehensive biomechanical modeling. As a retrospective, single-center study, the findings require cautious generalization. Nonetheless, the observed associations introduce a novel concept that advanced KOA represents a biomechanical predisposition to unstable ITFs, with important implications for surgical risk stratification.

Strengths of the Study: Despite these limitations, this investigation presents several strengths. It provides a focused analysis of the relationship between KOA severity and IHF stability using validated radiographic metrics. Inter-rater reliability was excellent for both KL grading and LWT measurement. The use of multivariable logistic regression and ROC curve analysis offers robust evidence that KL grade has strong discriminative ability for predicting fracture instability. Together, these findings underscore the clinical importance of assessing knee degeneration as an integral biomechanical factor in the evaluation and management of hip fractures.

## 5. Conclusions

In this cohort, KOA severity was significantly correlated with unstable ITF patterns. The KL grade emerged as an independent predictor of instability and demonstrated strong diagnostic performance. Although the causal pathways cannot be definitively determined from this retrospective design, the findings provide compelling evidence that degenerative alterations in the knee exert biomechanical effects on proximal femoral stability and fracture configuration. These insights highlight the clinical value of incorporating KOA severity into preoperative assessments—not only for improved risk stratification but also for optimizing surgical planning and rehabilitation strategies. Surgeons should consider KL grading as part of the standard evaluation for elderly patients with hip fractures, particularly when anticipating unstable morphologies. Future prospective studies are warranted to validate these findings and further clarify the mechanisms linking knee degeneration to hip fracture instability. Such work should integrate BMD assessments, characterization of fall mechanisms, gait analysis, and computational biomechanical modeling to establish causal pathways and inform targeted preventive or therapeutic interventions. Routine assessment of KOA severity may therefore provide surgeons with a practical, radiographic biomarker for predicting hip fracture stability and optimizing fixation strategy in elderly patients.

## Figures and Tables

**Table 1:** Baseline Characteristics of Patients with Stable vs. Unstable Intertrochanteric Hip Fractures.

Variable	Stable Fractures (n = 40)	Unstable Fractures (n = 98)	*p*-Value
Age, mean ± SD (years)	65.6 ± 21.4	75.2 ± 13.0	0.011
Male sex, %	72.5%	51.0%	0.021
BMI, mean ± SD	24.3 ± 3.9	24.1 ± 4.8	0.81
Hypertension, %	32.5%	41.8%	0.31
Diabetes mellitus, %	17.5%	22.4%	0.53
Heart disease, %	10.0%	16.3%	0.35
KL grade distribution	KL II: 57.5%	KL III–IV: 93.9%	<0.001

**Table 2:** Correlation Between KL Grade and Lateral Wall Thickness (LWT). Interpretation: An increase in KL grade is strongly associated with reduced LWT across all correlation methods.

Correlation Type	Coefficient (r/τ)	*p*-Value
Pearson correlation	−0.394	<0.001
Spearman correlation	−0.403	<0.001
Kendall’s tau	−0.316	<0.001

**Table 3:** Logistic Regression Analysis for Predictors of Fracture Instability. KL grade was the only independent predictor of fracture instability.

Variable	Odds Ratio (OR)	95% CI	*p*-Value
KL grade (per grade increase)	4.9	2.8–8.8	<0.001

**Table 4:** Diagnostic Performance of KL Grade for Predicting Unstable Fractures.

Metric	Value
Area under the ROC curve (AUC)	0.79 (95% CI: 0.70–0.88)
Optimal threshold (Youden Index)	KL ≥ III
Sensitivity	95%
Specificity	56%
Positive Predictive Value (PPV)	80.0%
Negative Predictive Value (NPV)	85.2%
Overall accuracy	84.1%

## Data Availability

Please contact the authors for data requests.
